# Theta Dynamics in Rat: Speed and Acceleration across the Septotemporal Axis

**DOI:** 10.1371/journal.pone.0097987

**Published:** 2014-05-19

**Authors:** Lauren L. Long, James R. Hinman, Chi-Ming Chen, Monty A. Escabi, James J. Chrobak

**Affiliations:** 1 Department of Psychology, University of Connecticut, Storrs, Connecticut, United States of America; 2 Biomedical Engineering, University of Connecticut, Storrs, Connecticut, United States of America; 3 Electrical and Computer Engineering, University of Connecticut, Storrs, Connecticut, United States of America; Centre national de la recherche scientifique, France

## Abstract

Theta (6–12 Hz) rhythmicity in the local field potential (LFP) reflects a clocking mechanism that brings physically isolated neurons together in time, allowing for the integration and segregation of distributed cell assemblies. Variation in the theta signal has been linked to locomotor speed, sensorimotor integration as well as cognitive processing. Previously, we have characterized the relationship between locomotor speed and theta power and how that relationship varies across the septotemporal (long) axis of the hippocampus (HPC). The current study investigated the relationship between whole body acceleration, deceleration and theta indices at CA1 and dentate gyrus (DG) sites along the septotemporal axis of the HPC in rats. Results indicate that whole body acceleration and deceleration predicts a significant amount of variability in the theta signal beyond variation in locomotor speed. Furthermore, deceleration was more predictive of variation in theta amplitude as compared to acceleration as rats traversed a linear track. Such findings highlight key variables that systematically predict the variability in the theta signal across the long axis of the HPC. A better understanding of the relative contribution of these quantifiable variables and their variation as a function of experience and environmental conditions should facilitate our understanding of the relationship between theta and sensorimotor/cognitive functions.

## Introduction

The laminar organization of the hippocampus (HPC) provides an optimal architecture for the generation of local field potentials (LFPs) such as theta or sharp waves [1]–[3]. These LFP signals reflect the summation of local excitatory and inhibitory synaptic potentials impinging upon the somatodendtric field of hippocampal neurons. The theta LFP (6–12 Hz) reflects synchronizing synaptic input impinging on relatively autonomous neurons, thus contributing to the integration and segregation of distributed network neurons into cell assemblies [2]–[10].

The theta signal has been linked to cognitive variables across several mammalian species [11]–[19] and variation in the signal can correlate directly with cognitive variables, such as the strength of encoding as evidenced by a relation to subsequent memory performance [20]–[23]. Historically, moment-by-moment variation in the amplitude and frequency of theta in the rodent HPC has been associated with locomotor speed and linked to sensorimotor/path integration [24]–[26]. Recent findings have highlighted significant variation in the amplitude and coherence of the theta signal across the septotemporal, areal or long axis of the HPC [27]–[30]. The latter is consistent with a large literature detailing functional and anatomical variation across the longitudinal (anteroposterior in humans) axis [31].

Early work investigating the behavioral correlates of the hippocampal theta signal observed its emergence during locomotion, specifically running speed of the rodent [32]–[36]. The increase in theta power as a function of running speed has been confirmed in subsequent studies [37]–[39], but recently has been shown to vary systematically across the long axis of the HPC [28], [30]. Most rodent studies examining the relationship between cognitive performance and theta involve spatial locomotion often along relatively fixed trajectories [12], [40]–[41] and can involve deceleration when animals are within regions of “choice” (often turns). Thus, a better understanding of the relative contribution of speed and acceleration to changes in theta should facilitate our understanding of how variations in theta relate to sensorimotor and/or cognitive processes. The current study demonstrates that whole body acceleration and deceleration of the rat was a significant predictor of theta amplitude over and above the influence of locomotor speed and that deceleration was much more predictive of theta amplitude than acceleration. The results are discussed with regards to variation in the relationship between theta and sensorimotor variables along the long axis of the HPC and their underlying neurobiological mechanism.

## Materials and Methods

### Overview

**Figure 1:**
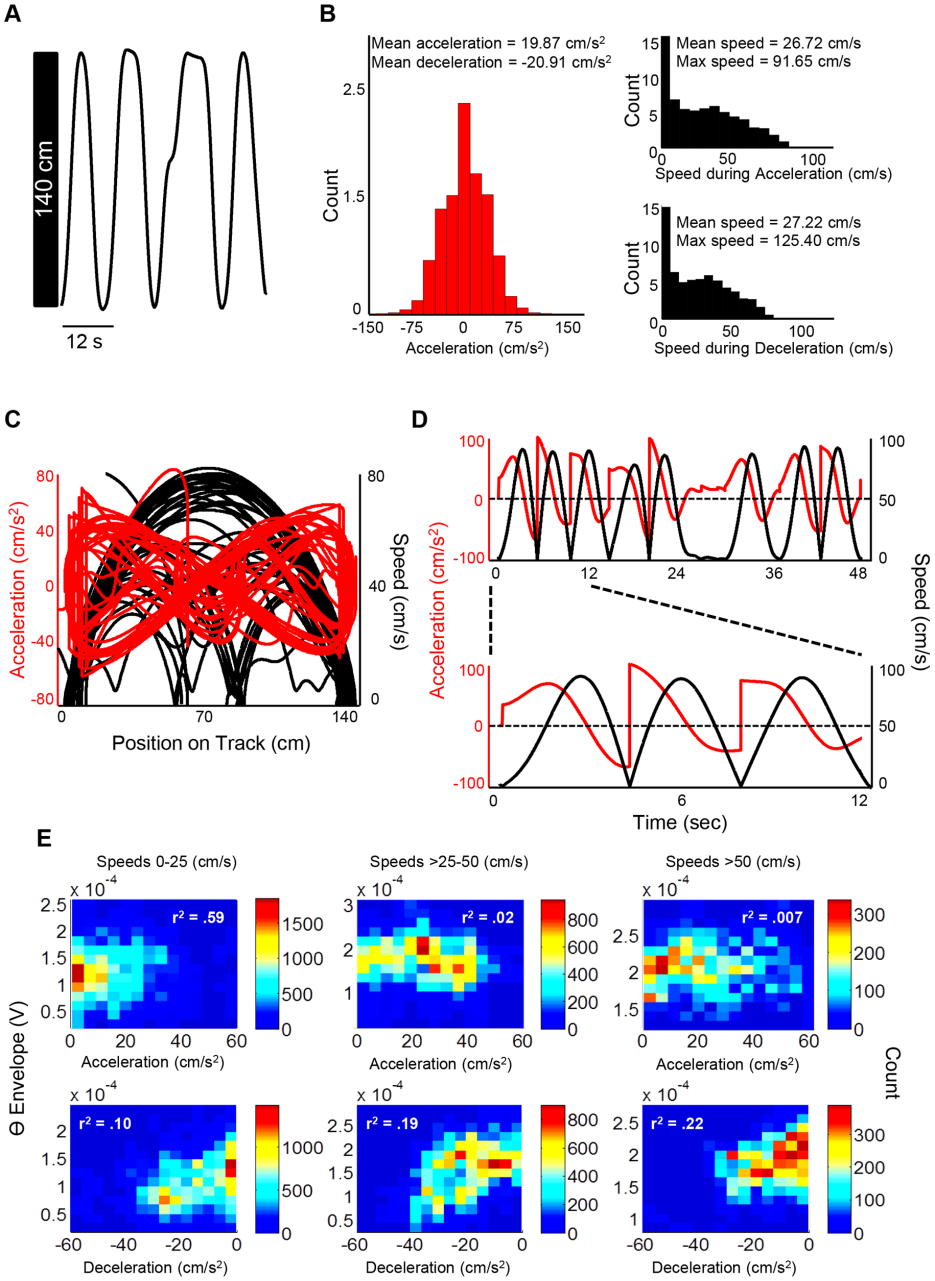
Methodological specifications. **A**: The rats' position on the 140 cm long maze (y-axis) over time (x-axis). 8 consecutive trials are shown. **B (left)**: Distribution of accelerations for all rats across all recording sessions. Max acceleration = 102.70 cm/s^2^; max deceleration = −105.74 cm/s^2^ (count units = ×10^5^). **B (right)**: Distribution of speeds for all rats across all recording sessions during acceleration and deceleration (count units = ×10^4^). **C**: The rats' speed (black) and acceleration (red) as a function of position on the maze for an entire recording session for one rat (∼5 minutes). Acceleration is shown in both running directions in order to emphasize the similar distribution of accelerations/decelerations. **D (top)**: Speed (black) and acceleration (red) as a function of time. 8 consecutive trials are shown in order to visualize the relationship between speed and acceleration/deceleration. **D (bottom)**: A closer look at the first 12 seconds of the top signals, now only the first 3 consecutive trials are shown. **E**: Relationship between acceleration and theta amplitude (top) and deceleration and theta amplitude (bottom) as a function of “low” and “high” speeds.

Data used in current analyses was collected as previously described in Hinman and colleagues (2011) [30] where rats were trained to run back and forth across a 140 cm linear track as illustrated in Fig. 1A. Key differences in the current data analyses relate to the use of data over the entire recording session (∼5 min/rat) that includes both periods of movements as well as non-movement (with speeds ranging from ∼0–125 cm/s). Thus, the current data-set includes a considerable range of accelerations and decelerations (see Fig. 1B for distribution of speed and acceleration/deceleration values). In this regard, the relationship between deceleration and theta amplitude controlling for speed (Fig. S1A) varied when we included data from the entire run session (blue) or removed more stationary epochs (red; t(26) = −3.108, *p* = .005; green: t(26) = 2.709, *p* = .012; Fig. S1A). Alternatively, the relationship between acceleration and theta amplitude controlling for speed did not vary when including all data or removing speeds less than 5 cm/s (Fig. S1B blue vs. red line; t(26) = −1.950, *p* = .062), but did vary when concurrently including a position and speed cutoff (Fig. S1B blue vs green line; t(36) = −2.359, *p* = .026). These data are not surprising given the restriction in the dynamic range of decelerations when removing the extremities of the track. Because the extremities of the track contain much of the dynamic variability in deceleration (as well as theta amplitude), removing such variation constrains the analysis and thus reduces the correlations.

Considering the well-known relationship between locomotor speed of the animal and theta amplitude [24]–[26], we sought to examine this relationship during acceleration and deceleration. The speed to theta amplitude relationship during acceleration and deceleration is significantly different than zero at all CA1 septotemporal extents (Fig. S1C; septal CA1 speed to amplitude during deceleration, faded blue bars t(4) = 9.321 *p* = .001 speed to amplitude during acceleration, faded red bars t(4) = 13.369 *p* = .0002; midseptotemporal deceleration t(13) = 4.779 *p* = .0004 acceleration t(13) = 6.433 *p* = .00002; temporal deceleration t(7) = 3.932 *p* = .006 acceleration t(7) = 3.989 *p* = .005), meaning that theta amplitude is significantly modulated by speed during epochs of acceleration and deceleration. Further, there is a significant difference in the modulation of theta amplitude by speed during acceleration and deceleration in only septal CA1 (Fig. S1C; septal CA1 t(4) = 3.015 *p* = .039; midseptotemporal t(13) = 1.633 *p* = .126 n.s.; temporal t(7) = 2.115 *p* = .072 n.s.), suggesting that there is a slight difference in the speed modulation of theta amplitude relationship during acceleration/deceleration.

### Animals and Surgical Procedures

#### Ethics Statement

All procedures performed were in strict accordance with the guidelines and regulations implemented by the University of Connecticut's Institutional Animal Care and Use Committee and NIH. The protocol was approved by the Institutional Animal Care and Use Committee of the University of Connecticut (Protocol Number: A12-014) and all efforts were made to minimize suffering.

Six Fisher-344 adult male rats, singly housed in a temperature/light controlled environment were used in the present study. Rats were anesthetized with a ketamine cocktail solution (4 ml/kg consisting of 25 mg/ml ketamine, 1.3 xylazine mg/ml, and 0.25 acepromazine mg/ml). A midline scalp incision was made, burr holes drilled through the skull over the HPC, and three –four electrode arrays were situated across the septotemporal axis of the HPC. All electrode arrays were comprised of four linearly spaced 50 µm tungsten wires (16 electrodes per animal; California Fine Wire Company, Grover Beach, CA). Electrode wire was arranged and separated by fused silica tubing (Polymicro Tubing, Phoenix, AZ), attached to female pins (Omnetics, Minneapolis, MN) and secured in a rectangular five by four pin array. Two stainless steel watch screws driven into the skull above the cerebellum served as indifferent and ground electrodes. Supplementary anchor screws were positioned anteriorly and the entire head-stage ensemble was fortified with dental acrylic. The surgical coordinates, where bregma was used as the reference point, were as follows: septal HPC (AP -3.0, ML 2.5, DV 3.0); midseptotemporal HPC (AP -5.0, ML 5.0, DV 5.0); temporal HPC (AP -6.5, ML 5.5, DV 7.0). Rats recovered for one week post-surgical procedure.

### Behavioral Measures, Electrophysiological Data Acquisition & Analyses

Animals were trained to run on a 140 cm linear track for a chocolate sprinkle food reward. Recordings required the animal to run 50 trials, where a single trial was denoted as a traversal from one end of the linear track to the other end. Five recording sessions where time was the only manipulation occurred within a single day. All data presented in the current analyses were from the first recording session as there is a systematic decrease in the theta signal as a function of repeated behavioral performance within a day [30].

Wide-band electrical activity was recorded (1–1894 Hz, 3787 samples/sec) using a Neuralynx data acquisition system (Bozeman, MT) and was down-sampled by a factor of 6 during offline analysis, thus changing the sampling rate to 631.167 samples/sec (Hz). The raw signal was bandpass filtered between 6 and 12 Hz and the Hilbert transform was computed on the bandpass filtered signal. In this regard, the instantaneous (631.167 samples/sec) theta envelope amplitude (magnitude of Hilbert transform) was obtained over time (See Fig. S2 for multiple examples of raw LFP signals along with corresponding filtered theta and envelope). Additionally, light emitting LEDs attached to the headstage were tracked by a camera (33 samples/sec, Hz) situated over the linear track, allowing for a record of the rats' position over time (Fig. 1A). The tracking data was up-sampled using a cubic spline interpolator (interp1 function in MATLAB) to 631.167 Hz in order to match the LFP data sampling rate. Speed was calculated by taking the finite difference between successive tracking (position) samples followed by a low-pass filter (cutoff = 0.25 Hz; see Fig. S3 for relationship between position, speed, acceleration, deceleration and theta amplitude at different time-scales) to minimize head movements and other movement related artifacts (see Fig. 1B for distribution of speed during acceleration and deceleration). The kinematic signals (e.g. speed, acceleration, deceleration) are primarily low-pass in nature such that the coherence between theta amplitude and the unfiltered biomechanical signals is maximal at frequencies less than 0.5 Hz (Fig. S3C/D). For this reason, we chose to low-pass filter (0.25 Hz) the kinematic signals in order to remove uncertainty by filtering out non-coherent, higher frequency signals in order to enhance the ability to observe correlations between theta and the relevant biomechanical signals. Position and velocity data were visualized as a state-space plot (Fig. 1C, black). Further, acceleration was calculated by taking the second-order finite difference with regards to position followed by the same low-pass filter applied to the speed signal (Fig. 1C, red). To visualize the relationship between speed and acceleration/deceleration, all indices were plotted as a function of time (Fig. 1D). All data analysis were performed using custom written programs in MATLAB (The MathWorks, Natick, MA), with additional statistical analysis computed in SPSS (IBM, Armonk, NY).

### Spectral Indices & Statistics

For each recording, theta envelopes were calculated as an instantaneous measure of theta amplitude. The instantaneous envelope amplitude from each electrode was subjected to a multiple regression analysis that included the speed, acceleration and the interaction between the two (speed x acceleration) in order to assess the relationship between locomotor speed, acceleration and theta amplitude. Thus, each electrode yielded a standardized regression coefficient value (beta, *β*) that assessed the linear association between speed, acceleration, and the interaction of theta envelope by speed/acceleration [42]. Beta coefficients standardize predictor variables such that their variances equal one. Further, beta coefficients describe how many standard deviations a response variable (in this case theta amplitude) will change with a one standard deviation increase in a given predictor variable (e.g., speed) [42]–[43]. Thus, beta coefficients describe which predictor variable has a greater effect on a given response variable, and each beta-value for a given electrode can be inserted into the model to accurately predict theta amplitude. A non-significant beta coefficient indicates that a predictor variable does not significantly contribute to explaining variability in the response variable. Assumptions met upon calculation of the multiple regression include independent, random, and normal distribution of residuals and a distinct lack of outliers. Importantly, multiple regression analysis is remarkably robust with regards to violating assumptions of normally distributed residuals [42]–[43].

Beta coefficients obtained from the multiple regression indicate which predictor has a greater effect on the response variable, but do not indicate if the predictor variables are correlated (co-vary; multicolinearity) with each other [42]–[43]. In order to partial out the contribution of one predictor variable to another, partial correlations were calculated (partialcorr function in MATLAB). Thus, squared partial correlations may be understood as the proportion of variance not associated with other predictor variables and that is associated with the predictor variable of interest [42]–[49]. If a predictor significantly contributes to explaining variability in the response variable, as indicated by a significant beta (β) coefficient in the multiple regression model (data not shown), that predictor was added to the partial correlation analysis. For a clearer interpretation, partial correlations inherently tend to interaction terms (e.g. speed x acceleration) as the relationship between theta amplitude and a given predictor variable (e.g. acceleration) is independent of variations attributed to speed. In this regard, partial correlations “partial out” variability in theta amplitude attributed to speed and the interaction of speed and acceleration by treating speed as a constant over all acceleration values. Thus, relationships between acceleration and theta amplitude are autonomous from variations in locomotor speed as well as variations in the interaction of acceleration and speed [42]–[49]. For the partial correlation, acceleration was divided into one of 2 categories 1) acceleration (positive acceleration) and 2) deceleration (negative acceleration). The corresponding theta amplitude was indexed for each acceleration category and was added to the partial correlation model. Since zero acceleration can be denoted as no movement (stopped at end of trial) or constant movement (e.g., no acceleration), these data points were not included in analyses. Furthermore, these data points have very little or no contribution to the overall model due to their extremely low values.

### Electrode Groupings & Statistics

Electrodes within each septotemporal extent of DG and CA1 were separately grouped in order to determine whether areal region had a mean partial correlation (speed/acceleration/deceleration) value that was different than zero using a single-sample t-test. A significant non-zero mean for a particular speed, acceleration and deceleration partial correlation value indicates that theta amplitude was significantly modulated by speed, acceleration and/or deceleration [50]. Furthermore, linear correlations were conducted on the partial correlation values (e.g., partial correlation between theta amplitude and acceleration) for areal regions accompanied by distance from the septal pole (millimeters) as an explanatory variable, allowing for the demonstration of whether speed and acceleration/deceleration modulation of theta amplitude varied across the septotemporal axis of CA1 and DG. Paired-sample t-tests were conducted to assess if there were significant differences in 1) relationships between acceleration, deceleration and theta amplitude and 2) modulations of theta amplitude by acceleration/deceleration in different hippocampal subregions (e.g., CA1 vs. DG).

### Histological Methodology

Animals were transcardially perfused with ice-cold saline followed by 4% paraformaldehyde in .1M phosphate buffer. Brains were sliced using a vibratome, mounted, and Nissl stained using thionin. Septotemporal distances between electrodes were verified by placing each electrode position on a flatmap representation of the HPC [51]. Each section of a flatmap represents ∼200 µm of tissue, and so fairly accurate approximations of the relative distance between electrodes could be determined by counting the number of sections between two electrodes. The most septal portion of the HPC represents 0 mm and serves as a reference for all electrodes. Septotemporal groupings were as follows: Septal: 0–3 mm; Midseptotemporal: 3.1–6 mm; Temporal: 6.1+ mm. Photomicrographs of electrode tracks were taken, digitized and prepared for presentation.

## Results

### Behavioral Performance

Data from the entire recording sessions was utilized in analysis, which included a wide distribution of speeds and accelerations values. The resulting dataset contained an average of 46.4+/−0.88 (SEM) trials (run from one end of the linear maze to the other end) per recording. The mean acceleration was 19.87 cm/s^2^ with a maximum acceleration of 102.70 cm/s^2^, while the mean deceleration was −20.91 cm/s^2^ with a maximum deceleration of −105.74 cm/s^2^ (Fig. 1B, red). Further, the distribution of speeds was discretized according to acceleration and deceleration. The mean speed during acceleration was 26.72 cm/s, with a maximum speed of 91.65 cm/s (Fig. 1B, black; top). Moreover, the mean speed during deceleration was 27.22 cm/s, with a maximum speed of 125.40 cm/s (Fig. 1B, black; bottom). Additionally, the relationship between acceleration/deceleration and theta amplitude is shown as a function of distinct speed categories (0–25 cm/s; >25–50 cm/s; >50 cm/s; Fig. 1E) and evidences a possible interaction between speed and acceleration such that the relationship between theta amplitude and acceleration appears to be maximal at low speeds (< = 25 cm/s) whereas the relationship between deceleration and theta amplitude is maximal at high speeds (>50 cm/s).

### Histology: Electrode Placements

**Figure 2:**
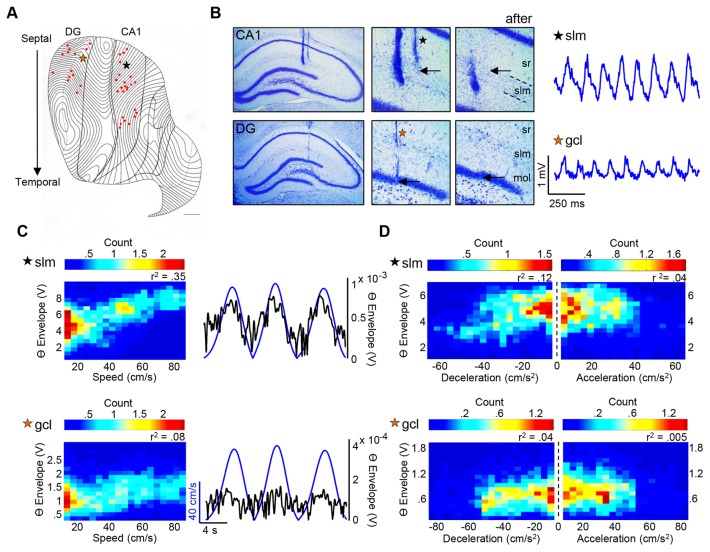
Electrode locations, corresponding theta traces & relationship between theta amplitude and speed/acceleration/deceleration. **A**: Flatmap representation of the hippocampal formation. Electrode placements are indicated as red dots. Each contour line represents a coronal section. Orange star denotes DG electrode as in B–D (bottom), while black star denotes CA1 electrode as in B–D (top). **B (top)**: Photomicrographs of a representative recording site in septal CA1. Middle photomicrograph shows a close-up (20×) of electrode tip, as denoted by the black arrow. The right photomicrograph depicts the next coronal section for verification that the electrode tract ends. The septal CA1 tract ends in slm. The raw, unfiltered LFP for representative CA1 slm electrode is shown. **B (bottom)**: Same as top (CA1), but for DG. The septal DG tract ends in the gcl. Theta trace for representative DG gcl electrode is shown. **C**: two-dimensional histogram (density plot) of the relationship between speed and theta amplitude for representative CA1 slm and DG gcl electrodes, as well as speed signal with overlaid theta trace. **D**: two-dimensional histograms for the relationship between theta amplitude and acceleration/deceleration for the same CA1 slm and DG gcl electrodes (all theta envelope units in 2D histograms = ×10^−4^; all count units = ×10^3^; all *p*-values<.0001). ***Abbreviations***: sr = stratum radiatum; slm = stratum lacunosum moleculare; mol = molecular layer; gcl = granule cell layer.

Histological verification was as previously reported [30] with the majority of sites positioned in stratum radiatum of CA1 (N = 27 sites); additional sites within CA1 spanned from the ventral aspect of stratum pyramidale to stratum lacunosum moleculare. DG (N = 15) sites were mainly positioned in stratum granulosum or stratum moleculare. With regards to septotemporal position, CA1 sites spanned from 1.3–7.6 mm along the long axis, while DG sites were more narrow and spanned from 1.4–4.8 mm (septal and mid-septotemporal regions, Fig. 2A). Theta amplitude varied as a function of laminar position in septal HPC, as has been well-documented elsewhere [6]. Photomicrographs show septal CA1 and septal DG (Fig. 2B, top and bottom, respectively) placements and the following coronal section after the end of the electrode tip in order to confirm its termination. The relationship between speed, acceleration/deceleration and theta amplitude is shown for representative electrodes using 2-dimensional histograms (Fig. 2C and D) along with the relationship between theta (black trace) and speed (blue trace; Fig. 2C). Two-dimensional histograms represent the joint distribution of variables X and Y (e.g., speed and theta amplitude, respectively) and are thus color-coded according to the number of occurrences where Y (e.g., theta amplitude) is a particular value at a given X (e.g., speed, acceleration, deceleration) value. Warmer colors (e.g., red) signify that there are a higher number of occurrences where Y is a particular value at a given X value. For a clearer interpretation, two-dimensional histograms can best be understood and visualized as a scatter-plot with an overlaid grid, where the numbers of points are counted within each pixel of the grid and represented on a color scale.

### Acceleration and Deceleration Predict Variation in Theta Amplitude

#### CA1

**Figure 3:**
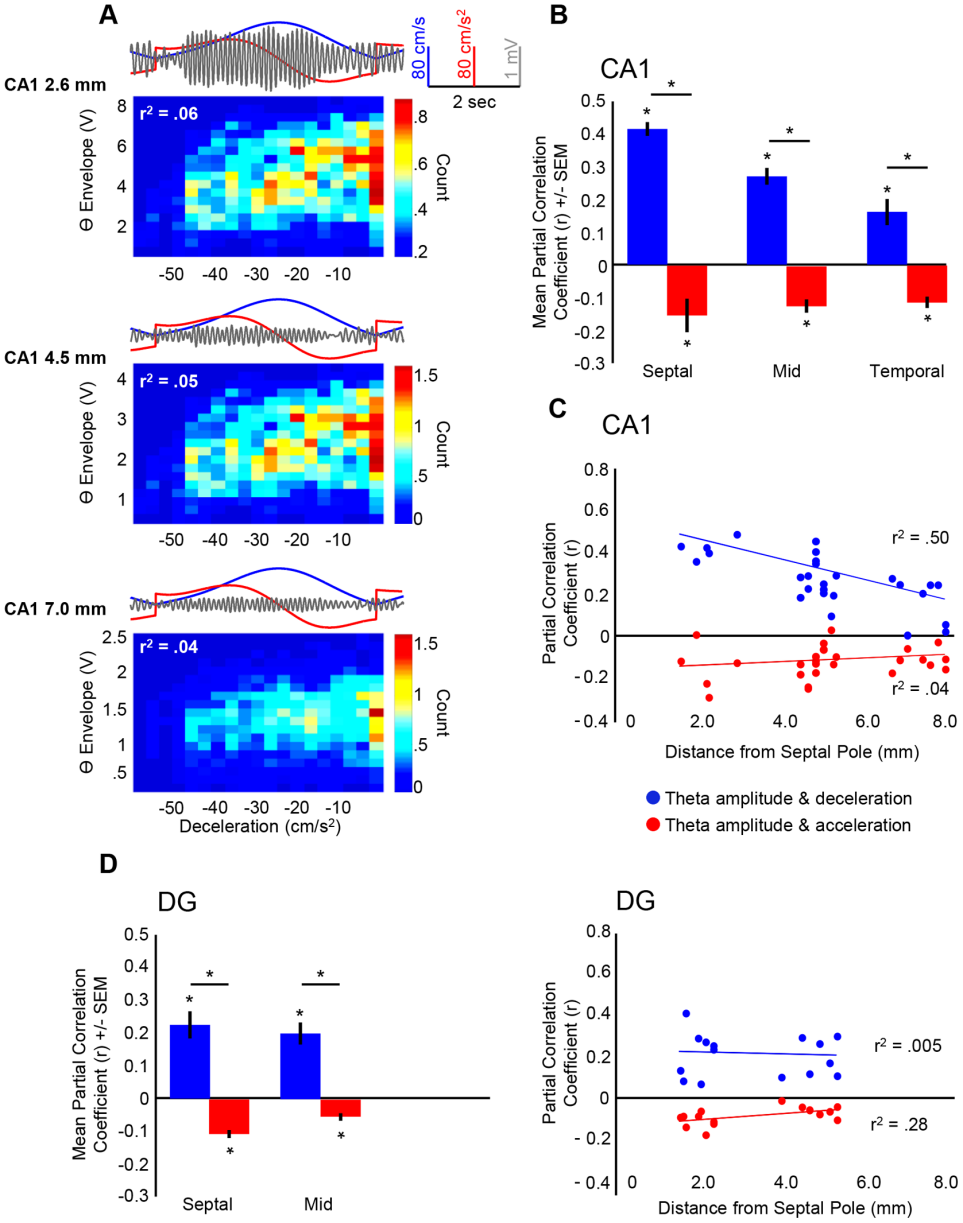
Relationship between acceleration, deceleration and theta amplitude. **A**: two-dimensional histograms for the relationship between deceleration and theta amplitude, with corresponding filtered theta, speed and acceleration traces for simultaneously recorded CA1 electrodes. All theta envelope units = ×10^−4^; all count units = ×10^3^. **B**: Electrodes were grouped according to septotemporal position. Mean partial correlation coefficients (controlling for speed) are shown for the relationship between deceleration (blue bar) and theta amplitude as well as for acceleration (red bar) and theta amplitude for CA1. As can be seen, when acceleration is separated into its positive and negative constituents, a differential relationship emerges such that deceleration is more predictive of theta amplitude as compared to acceleration. Theta amplitude was significantly modulated by both acceleration and deceleration across the entirety of the hippocampus for CA1. Additionally, deceleration explained more of the variability in theta amplitude across the entirety of CA1 axis. **C**: Partial correlation coefficients for the relationship between deceleration and theta amplitude (blue circles) and acceleration and theta amplitude (red circles) as a function of distance from the septal pole for CA1. Each dot represents the partial correlation coefficient between each index (acceleration, deceleration) and theta amplitude plotted as a function of distance from the septal pole. The relationship between deceleration and theta amplitude decreased across the septotemporal axis of CA1. **D**: Same as A, but for DG. Theta amplitude was significantly modulated by both acceleration and deceleration at septal and midseptotemporal DG sites. Further, deceleration explained more of the variability in theta amplitude than acceleration at DG sites.

**Figure 4:**
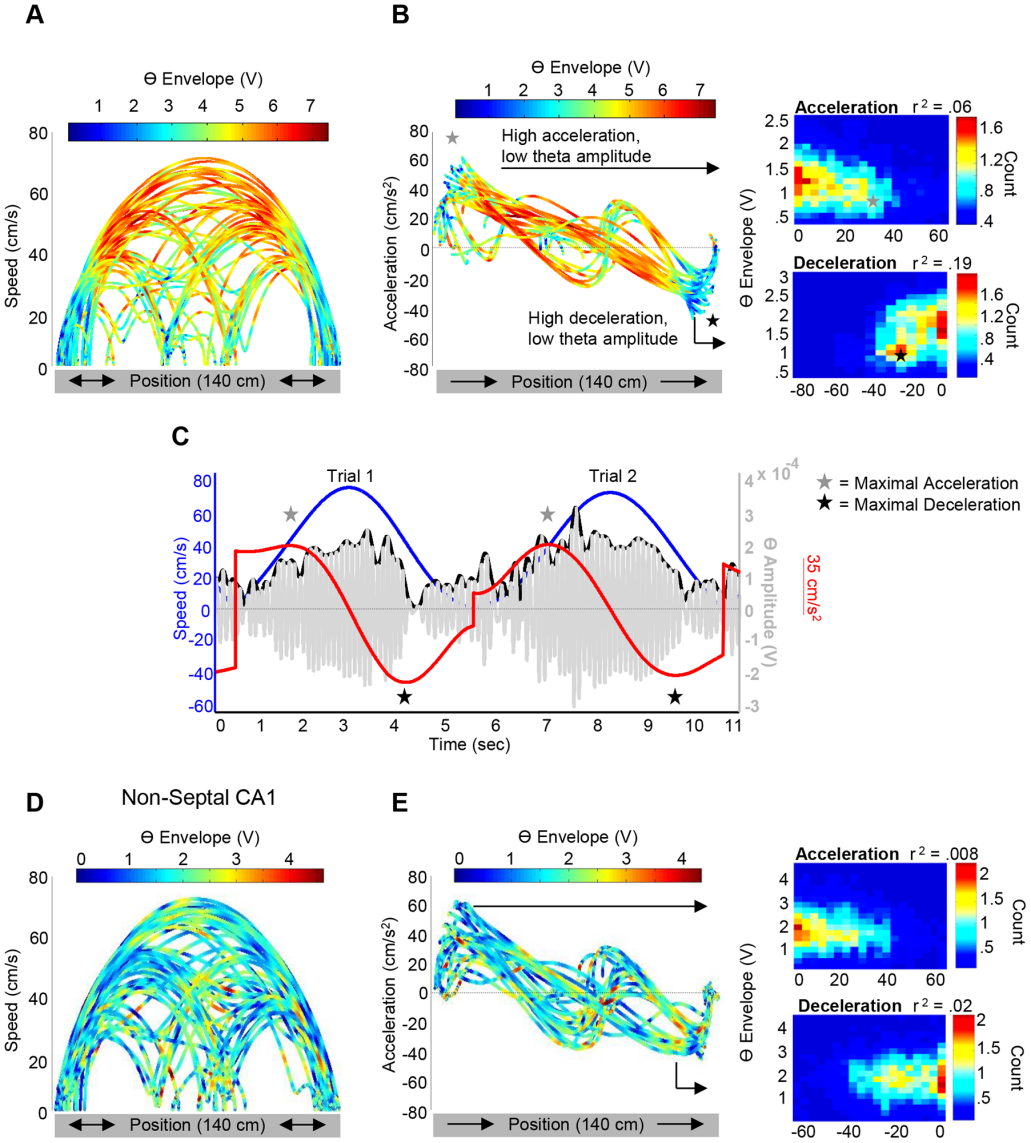
Speed, acceleration/deceleration and septal theta amplitude as a function of position on the maze. **A**: Three-dimensional scatterplot rotated to a specific view showing the relationship between position on 140-cm maze (x-axis), speed (y-axis), and color-coded for theta amplitude. As can be seen, with increasing and maximal speeds (centered in the middle of the maze) theta amplitude increases. **B (left)**: Same as A, but for acceleration in one direction (rat moving from left to right) as denoted by the black arrows on the x-axis. At high accelerations and high decelerations theta amplitude is low and increases in amplitude at less extreme accelerations. Gray star denotes high accelerations and low theta amplitude, while the black star denotes high decelerations and low theta amplitude. **B (right)**: Two-dimensional histograms depicting the relationship between acceleration and theta amplitude (top) and deceleration and theta amplitude (bottom). **C**: Filtered theta signal (gray) and theta envelope (black) plotted along with speed (blue) and acceleration (red). As can be seen, there is a sharp reduction of the theta amplitude at extreme accelerations and decelerations, and is more pronounced at high decelerations, as represented by the three-dimensional scatter plot and two-dimensional histograms in B. Gray stars represent time points of maximal acceleration, while black stars represent points of maximal deceleration. **D and E**: Same as A and B, but for a non-septal electrode. (All theta envelope units = ×10^−4^; all count units = ×10^3^).

Theta amplitude was modulated by both acceleration and deceleration, controlling for speed (partial correlations), at all CA1 sites along the long axis of the HPC as can be seen by three simultaneously recorded electrodes (Fig. 3A for deceleration). The mean partial correlation coefficients for all CA1 electrodes across the long axis were significantly different than zero for both acceleration (Fig. 3B red bars; septal: t(4) = −2.98, *p* = .041; midseptotemporal: t(13) = −5.912, *p*<.0001; temporal: t(7) = −6.464, *p* = .0003) and deceleration (Fig. 3B blue bars; septal: t(4) = 19.44, *p*<.00005; midseptotemporal: t(13) = 10.619, *p*<.0001; temporal: t(7) = 3.986, *p* = .005). Importantly, there was a significant difference in the modulation of theta amplitude by acceleration and deceleration with deceleration explaining ∼16% of the variability in septal CA1, while acceleration explained only ∼2% (septal: t(4) = −9.453, *p* = .001; midseptotemporal: t(13) = −10.399, *p*<.0001; temporal: t(7) = −5.891, *p* = .001). It's important to note that the relationship between locomotor indices and theta amplitude has been demonstrated to depend upon the time-scale of analysis [52]. In order to address this concern, we computed power spectral density, coherence and partial correlation coefficients between theta amplitude and locomotor indices as a function of different locomotor speed filter cut-offs (Fig. S3). While acceleration and deceleration predicted theta variability along the entire septotemporal extent of the HPC, there were differences across the long axis (see Fig. 3C and Fig. 4 for septal and non-septal electrodes). As is evident in Fig. 3C and 4, the relationship between deceleration and theta amplitude diminished across the long axis of CA1 (Fig. 3C blue circles; r = −.709, *p*<.00005, (r^2^ = .5)), while the relationship between acceleration and theta amplitude remains relatively constant (Fig. 3C red circles; non-significant r = .216, *p* = .279, (r^2^ = .04)). Each dot represents the partial correlation coefficient between each index (acceleration, deceleration) and theta amplitude plotted as a function of distance from the septal pole. For a more elaborate representation, Fig. 4 evidences the relationship between speed, acceleration/deceleration and theta amplitude as a function of position on maze for a septal (Fig. 4A and B) and non-septal CA1 (Fig. 4D and E) electrode and represented as a 3-dimensional scatterplot. Three-dimensional scatterplots represent the relationship between, for example, position on 140-cm maze (x-axis), speed (y-axis), and color-coded for theta amplitude. Importantly, presented three-dimensional scatterplots have been rotated to focus on a specific view and can thus be interpreted as a density plot. As evidenced in Fig. 4, a sharp reduction of theta amplitude appears at high accelerations and decelerations, although more prominent at high decelerations (see Discussion section). Furthermore, representative theta, speed, and acceleration signals are plotted for visualization of such relationship (see Discussion section for further explication).

#### DG

Although fewer electrodes were positioned across the DG areal axis, DG sites exhibited a similar relationship between acceleration, deceleration and theta amplitude as compared to CA1, where theta amplitude was modulated by both acceleration and deceleration at septal and midseptotemporal extents (Fig. 3D, left, acceleration: red bars and deceleration: blue bars; septal acceleration: t(7) = −8.102, *p* = .0008; septal deceleration: t(7) = 5.45, *p* = .001; midseptotemporal acceleration: t(6) = −4.455, *p* = .004; midseptotemporal deceleration: t(6) = 5.884, *p* = .001). In DG, deceleration predicted more variability in theta amplitude than acceleration (Fig. 3D, left; septal: t(7) = −6.484, *p* = .0003; midseptotemporal: t(6) = −5.915, *p* = .001). Unlike CA1, the relationship between deceleration and theta amplitude did not decrease across the long axis of DG (Fig. 3D, right blue circles; non-significant r = −.074, *p* = .792, (r^2^ = .005)), although lack of temporal DG placements could explain the current results.

#### CA1 vs. DG

Additionally, there was a significant difference in modulation of theta amplitude by acceleration at midseptotemporal CA1 and DG sites, such that acceleration explained more of the variability in theta amplitude at midseptotemporal CA1 sites as compared to midseptotemporal DG sites (Fig. 3B and D red bars: t(19) = −2.312, *p* = .032), while septal extents displayed no differences between DG and CA1 with regards to modulation of theta amplitude by acceleration (Fig. 3B and D red bars: t(11) = −1.137, *p* = .280). There was a significant difference in CA1 and DG in modulation of theta amplitude by deceleration at septal extents only (Fig. 3B and D blue bars: t(11) = 3.525, *p* = .005), while CA1 and DG midseptotemporal extents displayed no difference (Fig. 3B and D blue bars: t(19) = 1.68, *p* = .109 non-significant findings).

### Summary

Overall these data suggest that there was a differential relationship between theta amplitude and acceleration/deceleration, with deceleration explaining more of the variability in theta amplitude as compared to acceleration over and above the influence of locomotor speed. Notably, deceleration explains ∼16% of variability in theta amplitude in septal CA1. Moreover, the relationship between deceleration and theta amplitude decreases across the septotemporal axis of CA1, while the relationship between acceleration and theta amplitude remains constant. The effect of acceleration/deceleration on theta amplitude in DG follows a similar pattern with that of CA1; however, we found little support for differences across the septotemporal axis.

## Discussion

Network activation as measured by LFP theta signals in the HPC can be used as a tool to better understand moment-by-moment dynamics across the septotemporal axis of the HPC. Similar to analysis of variations in the blood-oxygen-dependent (BOLD) signal used in functional neuroimaging [53]–[54], detailed analysis of theta signal reveals the engagement of distributed neural circuits in relation to ongoing sensorimotor experience as well as cognitive operations [11], [12], [15]–[20], [55]. The theta signal is fairly coherent across both the laminar and septotemporal axis of the HPC during a variety of theta states [3], [6], [29], however on a moment-to-moment basis there are significant differences in the amplitude of the signal in relation to both sensorimotor and cognitive variables [24], [30], [55]–[57]. The present research demonstrates that acceleration and deceleration, over and above locomotor speed, significantly contribute to predicting variability in theta amplitude. Second, it is quite clear that deceleration predicts more of the variability in theta amplitude (∼16% in septal CA1), while acceleration had a relatively minimal contribution in both DG and CA1 (∼2% in septal CA1). Further, the relationship between deceleration and theta amplitude decreased across the long axis of the HPC in CA1. The current findings highlight variability in theta signal across the long axis of the HPC and evidence a sharp reduction of theta amplitude (details below) in the septal HPC in relation to deceleration, which accompanies the termination of locomotion.

### Suppression of Theta Amplitude at High Accelerations/Decelerations

The present data illustrates a sharp reduction of theta amplitude that can be quantitatively related to both rapid acceleration and deceleration, but which was more prominent during deceleration that occurs at the termination of locomotion (Fig. 4B). This observation is consistent with that presented by Wyble and colleagues (2004) [25] where a sharp decrease in theta power (240–400 milliseconds) precedes the cessation of locomotor activity. In that study, rats shuttled between two ends of a linear track for food reward that was offered at only one end of the track. A prominent decrease in theta was observed at the baited end of the track, whereas power at the non-baited end of the track remained relatively constant. A variety of studies demonstrate a decline in theta amplitude prior to “expected sensory events that terminate approach” [26] in the septal HPC. The current findings confirm this prominent decline in theta as the rat decelerates and evidence that this suppression of theta amplitude progressively decreases in magnitude at CA1 sites along the long axis (Fig. 4E).

The current findings are consistent with a relationship between theta and the initiation and termination of voluntary motor acts within the septal HPC [24], [32], [58]. Bland and Oddie (2001) suggest that theta as manifested by hippocampal and associated structures functions to provide “voluntary motor systems with continually updated feedback on their performance relative to changing environmental (sensory) conditions” [24]. This general theoretical framework is supported by the underlying anatomy of hippocampal circuits that link multimodal associative cortices to ventral basal ganglia circuits [59]–[61]; the latter modulating voluntary behavior in relation to prefrontal cortical inputs. Several findings evidence theta synchrony between prefrontal cortex and the HPC with variability in coherence related to behavior and/or cognition [57], [62]–[64]. Notably the output of the HPC exhibits septotemporal (areal) variability [61]. A challenge for future studies is to 1) encapsulate variability in the theta signal across the long axis, 2) determine how and when theta synchrony links distributed networks across the forebrain, and 3) integrate that variability with emergent functions.

### Sensorimotor Versus Memory and Cognition

Many studies of theta have focused on the septal HPC in the rat and the prominent relationship between running speed and theta amplitude/frequency [24], [30]. Montgomery and colleagues (2009) [12] point out that while theta power has often been associated with the speed and/or acceleration of movement the “robustness of this correlation varies extensively across studies.” These authors highlight reports that “contextual effects such as running to or away from reward, motivation, or other task parameters account for as much or more of the variability in theta indices than speed and acceleration” [55]. Given those results, it is important to note that vertical head (bobbing) movements could co-vary with speed and acceleration/deceleration [65], while the relationship between acceleration/deceleration and theta amplitude possibly differs as a function of speed (see Fig. 1E). Moreover, the current dataset involves highly stereotyped behavior such that the current results may not hold in paradigms involving less anticipation of food reward, such as open field foraging behavior. Our recent studies have focused on theta activity as rats shuttle back and forth across a linear track and we have highlighted the following findings which evidence that both sensorimotor variables as well as “contextual” parameters contribute to the variability in theta. Briefly, locomotor speed and deceleration predict considerable variability in theta [30](as well as current findings) although the relationship decreases prominently with distance from the septal pole of the HPC. Importantly, habituation or repeated exposure to the same task in the same environment decreases theta amplitude most prominently at progressively more temporal HPC sites [30]. The latter is consistent with the noted intermittency in hippocampal theta reported by Royer and colleagues (2010) [66]. It appears the mechanisms that generate theta in the more temporal aspects of the HPC diminish upon repeated exposure to the same sensory environment or repetition of voluntary motor activity. Further, spatial novelty or running (linear maze) in a novel space increases the amplitude of theta throughout the septotemporal extent of the HPC, independent of running speed [56]. These and numerous other findings illustrate that changes in theta synchrony vary predictably with changing environmental (sensory) conditions as well as alterations in the pattern of voluntary motor activity, both of which would support the neural processes underlying cognitive performance particularly in spatial memory tasks [12], [55].

### Where Does the Locomotor Signal Originate?

“Consummatory” behaviors (e.g., chewing, drinking), immobility and slow wave sleep are associated with irregular hippocampal activity including delta waves and hippocampal sharp waves [67], while body movements (e.g., walking, running, lever-pressing) and rapid eye-movement sleep (REM sleep) are associated with hippocampal theta. The emergence of the theta signal in the HPC involves wholesale changes in a large network of neurons that minimally includes various brainstem afferents [68]– switching the dynamics of the medial septum into a theta-generating mode [68]. Typically, increases in medial septal input strengths are associated with increases in theta frequency [69]–[70]. It should be noted that brainstem afferents do not directly engage medial septal networks into a theta dynamic as septal under-cutting (deafferenation) alone can result in HPC theta [71]. Multiple interacting brainstem and hypothalamic circuits impinging largely on medial septal afferents provide an integrative switching mechanism that leads to the emergence of hippocampal theta rhythm.

Thus, sensory input (e.g., somatosensory) consequent to locomotion could modulate hippocampal theta activity by means of multiple sources. Medial thalamic areas [72] are thought to be important for the initiation of voluntary movements, such as walking, running and avoidance behaviors [73]. Furthermore, the vestibular system is implicated in stabilization of place cells [74] and spatial memory [75] where disruptions to the vestibular system produce decreases in theta indices [76]. Moreover, proprioceptive, visual and motor information can indirectly reach the HPC through the medial septum and/or entorhinal cortex [76]. More than likely, speed and acceleration information reaches the HPC through the dynamic interaction of multiple systems related to motor and sensory phenomena. Further, given the knowledge that slower frequency oscillations (e.g., theta) are generated by the recruitment of larger pools of neurons [77]–[78], it is likely that the neural “topography” of voluntary movements (such as running), is highly complicated and integrated compared to that of automatic movements [24].

### Summary

The findings of the current research highlight the importance of controlling for locomotor indices when attempting to relate theta indices to cognitive operations [12], [40]–[41], particularly in experimental paradigms that involve manipulations (e.g., behavioral choices, pharmacologic), sensorimotor variables and correlated changes in theta spectral indices. The present findings demonstrate predictable changes in theta LFP signals in relation to acceleration and deceleration and how that relationship changes along the septotemporal axis of the HPC.

## Supporting Information

Figure S1 **Relationship between deceleration, acceleration and theta amplitude during points of movement vs. non-movement.** **A**: Partial correlation coefficients for the relationship between deceleration and theta amplitude (controlling for speed) with inclusion of all data (∼0 to −125 cm/s; points of movement/non-movement, blue circles), the relationship between deceleration and theta amplitude with removal of non-movement epochs (Data>5 cm/s, red circles), as well as the relationship between deceleration and theta amplitude with a position and a speed cut-off (inclusion of Data>5 cm/s and 10 % position cut-off on both ends of maze, green circles) and all plotted as a function of distance from the septal pole. **B**: Same as A, but for acceleration. **C**: Mean partial correlations for the relationship between speed and theta amplitude controlling for deceleration (faded blue bar) and acceleration (faded red bar) for CA1.
(TIF)

Figure S2 **Examples of raw LFP and corresponding filtered theta and envelope.** **A**: Raw LFP traces for three different septal electrodes and their corresponding filtered theta signals (black, 6–12 Hz) and envelopes (red). **B and C**: Same as A, but for midseptotemporal and temporal extents, respectively.
(TIF)

Figure S3 **Relationship between locomotor indices and theta amplitude at different time-scales.** **A**: An example of an individual animal's unfiltered (blue) and filtered (red) position on maze over the length of the entire recording (time) with different filtering cut-offs (0.125, 0.25, 0.5, 1.0, 2.0 Hz). As can be seen the unfiltered and filtered position trajectories are highly similar. Furthermore, the unfiltered position trajectory is primarily low-frequency, such that high frequency components are absent. **B**: An example of an individual animal's unfiltered position (x-axis) plotted by its filtered position (y-axis) for each frequency cut-off. As can be seen, the filtered and unfiltered position trajectories are highly correlated with each other suggesting that our filter cut-off is accounting for much of the variability in the unfiltered position trajectory. **C (left)**: Power spectral density with removal of mean (“DC” component; red, left) for unfiltered position data (top, left, red) and unfiltered locomotor speed (bottom, left, red) and averaged across all animals (n = 6). **C (right)**: Coherence (gray, right) with removal of mean between filtered theta envelope (6–12 Hz) and unfiltered position data (top, right) and unfiltered locomotor speed (bottom, right) and averaged across all electrodes (n = 27). As can be seen, locomotor speed is a band-pass/low-pass function. **D (left)**: Same as C (left) but for unfiltered acceleration (top, left) and unfiltered deceleration (bottom, left). **D (right)**: Same as C (right) but for unfiltered acceleration (top, right) and unfiltered deceleration (bottom, right). E: Mean partial correlation coefficient between speed and theta amplitude controlling for acceleration (left) and deceleration (right) for all CA1 electrodes across the septotemporal axis and plotted as a function of different filter cut-offs. **F**: Same as E, but for the relationship between acceleration (left)/deceleration (right) and theta amplitude controlling for speed. As can be seen, the relationship between speed, acceleration/deceleration and theta amplitude are clearly band-pass/low-pass filter functions with higher frequency filter cut-offs accruing more noise and ultimately reducing correlations.
(TIF)
