# Supplementary figures and images for: Theta Dynamics in Rat: Speed and Acceleration across the Septotemporal Axis
Source: PLoS One. 2014 May 19;9(5):e97987. doi: 10.1371/journal.pone.0097987 (PMC4026415; doi:10.1371/journal.pone.0097987)

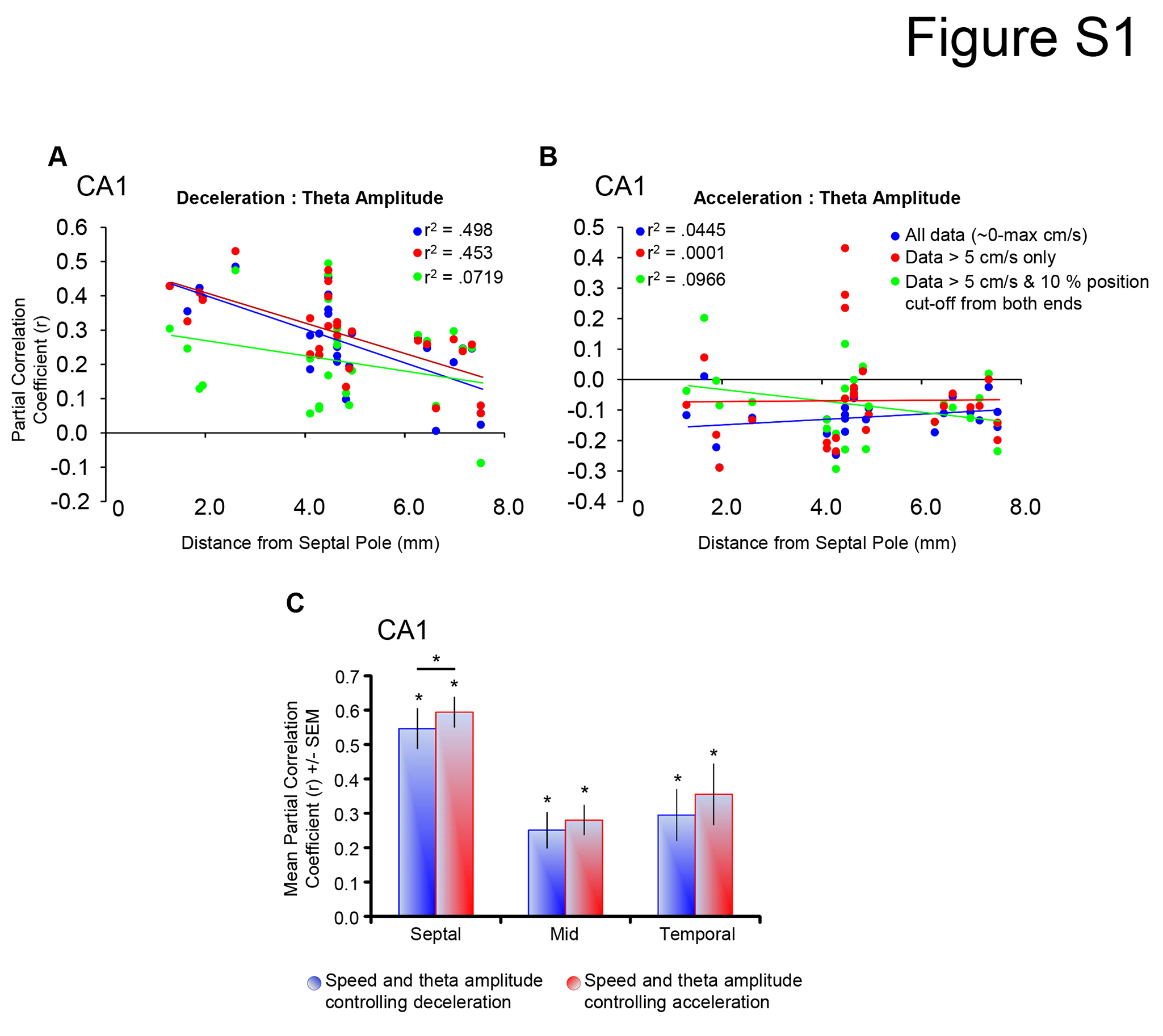

Supplement: Figure S1 — Relationship between deceleration, acceleration and theta amplitude during points of movement vs. non-movement. A: Partial correlation coefficients for the relationship between deceleration and theta amplitude (controlling for speed) with inclusion of all data (∼0 to −125 cm/s; points of movement/non-movement, blue circles), the relationship between deceleration and theta amplitude with removal of non-movement epochs (Data>5 cm/s, red circles), as well as the relationship between deceleration and theta amplitude with a position and a speed cut-off (inclusion of Data>5 cm/s and 10 % position cut-off on both ends of maze, green circles) and all plotted as a function of distance from the septal pole. B: Same as A, but for acceleration. C: Mean partial correlations for the relationship between speed and theta amplitude controlling for deceleration (faded blue bar) and acceleration (faded red bar) for CA1. (TIF) [file pone.0097987.s001.tif]

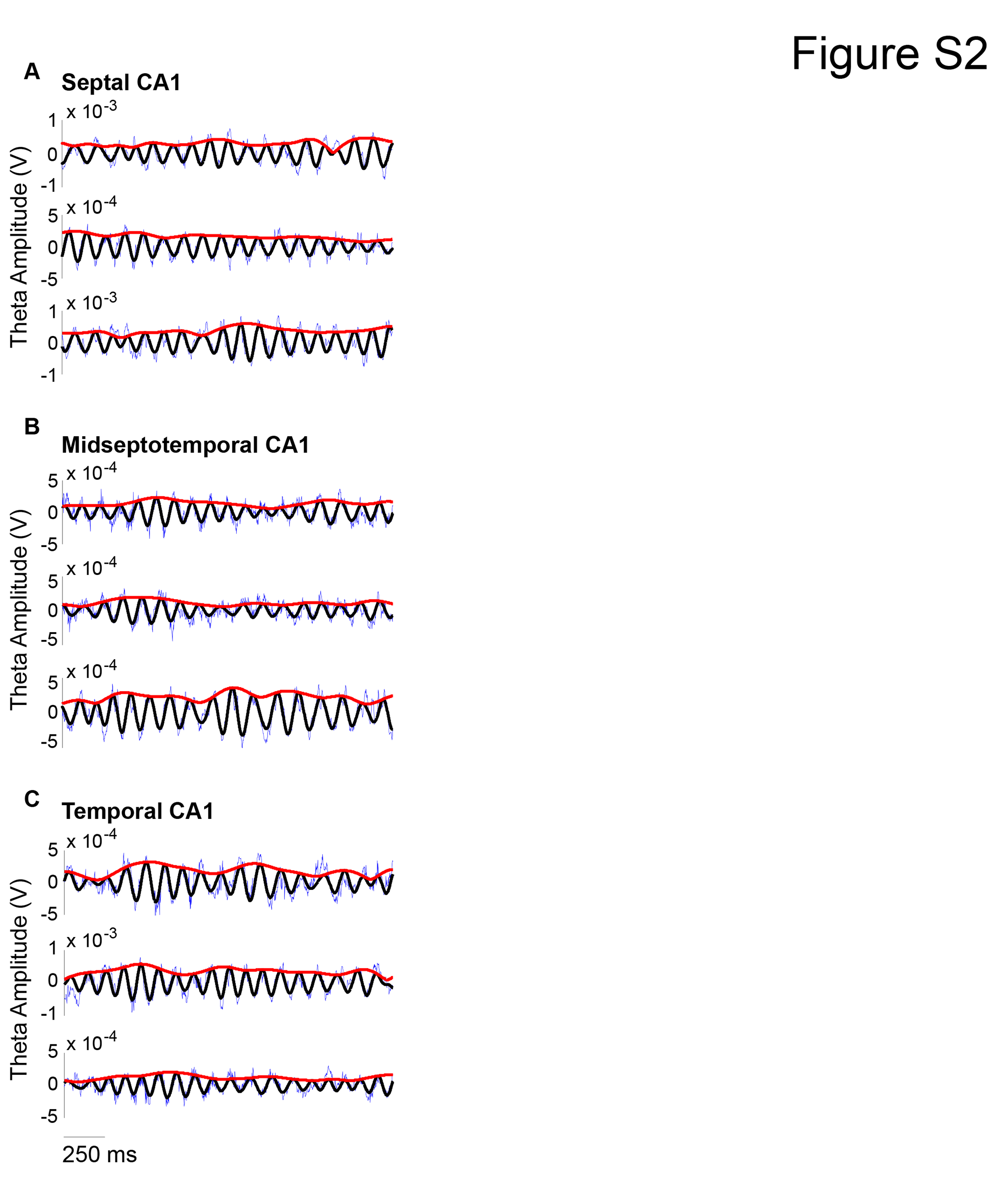

Supplement: Figure S2 — Examples of raw LFP and corresponding filtered theta and envelope. A: Raw LFP traces for three different septal electrodes and their corresponding filtered theta signals (black, 6–12 Hz) and envelopes (red). B and C: Same as A, but for midseptotemporal and temporal extents, respectively. (TIF) [file pone.0097987.s002.tif]

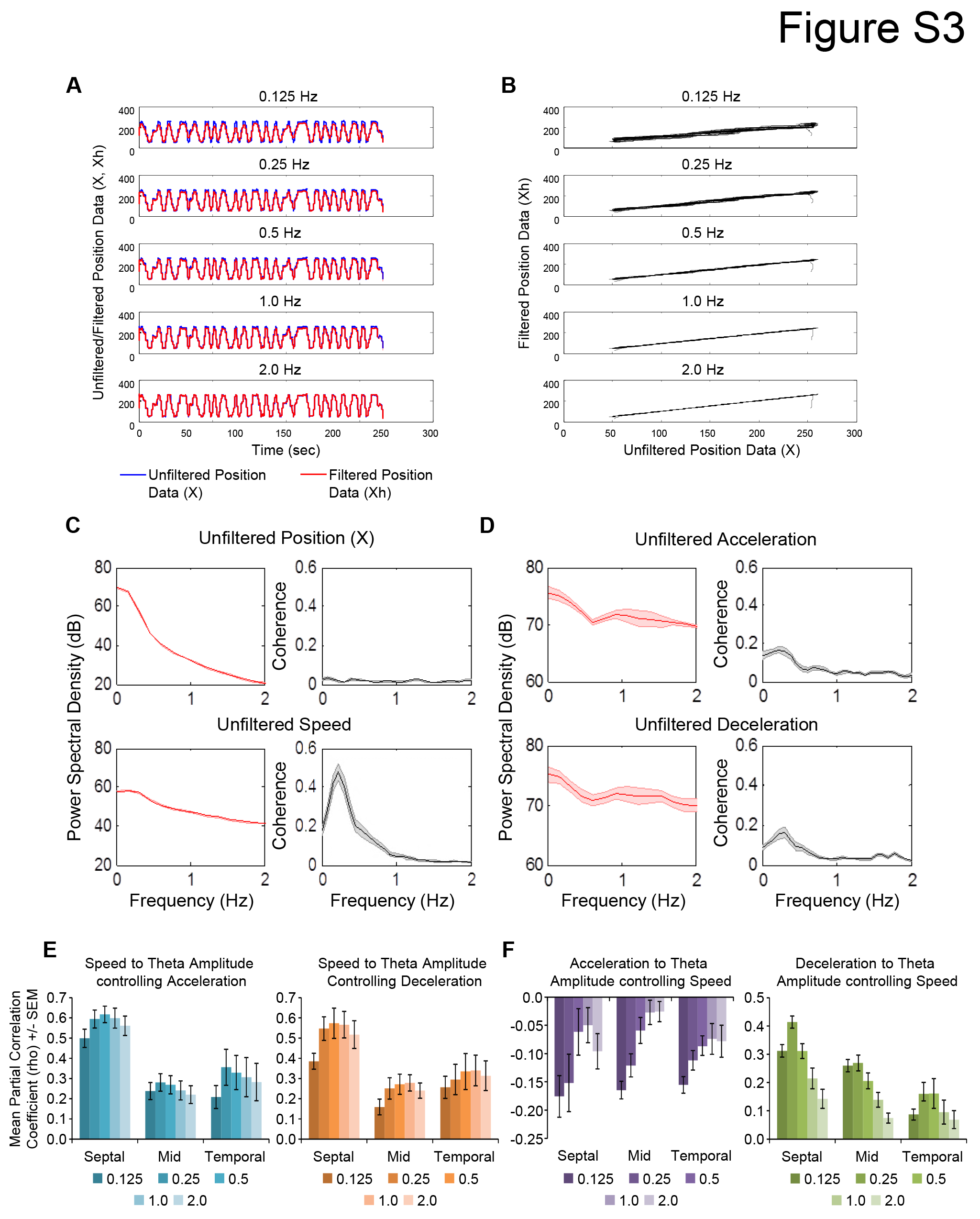

Supplement: Figure S3 — Relationship between locomotor indices and theta amplitude at different time-scales. A: An example of an individual animal's unfiltered (blue) and filtered (red) position on maze over the length of the entire recording (time) with different filtering cut-offs (0.125, 0.25, 0.5, 1.0, 2.0 Hz). As can be seen the unfiltered and filtered position trajectories are highly similar. Furthermore, the unfiltered position trajectory is primarily low-frequency, such that high frequency components are absent. B: An example of an individual animal's unfiltered position (x-axis) plotted by its filtered position (y-axis) for each frequency cut-off. As can be seen, the filtered and unfiltered position trajectories are highly correlated with each other suggesting that our filter cut-off is accounting for much of the variability in the unfiltered position trajectory. C (left): Power spectral density with removal of mean (“DC” component; red, left) for unfiltered position data (top, left, red) and unfiltered locomotor speed (bottom, left, red) and averaged across all animals (n = 6). C (right): Coherence (gray, right) with removal of mean between filtered theta envelope (6–12 Hz) and unfiltered position data (top, right) and unfiltered locomotor speed (bottom, right) and averaged across all electrodes (n = 27). As can be seen, locomotor speed is a band-pass/low-pass function. D (left): Same as C (left) but for unfiltered acceleration (top, left) and unfiltered deceleration (bottom, left). D (right): Same as C (right) but for unfiltered acceleration (top, right) and unfiltered deceleration (bottom, right). E: Mean partial correlation coefficient between speed and theta amplitude controlling for acceleration (left) and deceleration (right) for all CA1 electrodes across the septotemporal axis and plotted as a function of different filter cut-offs. F: Same as E, but for the relationship between acceleration (left)/deceleration (right) and theta amplitude controlling for speed. As can be se [file pone.0097987.s003.tif]
